# Long-Term Outcomes and Quality of Life of High-Risk Neuroblastoma Patients Treated with a Multimodal Treatment Including Anti-GD2 Immunotherapy: A Retrospective Cohort Study

**DOI:** 10.3390/cancers17010149

**Published:** 2025-01-05

**Authors:** Tim Flaadt, Jonas Rehm, Thorsten Simon, Barbara Hero, Ruth L. Ladenstein, Holger N. Lode, Desiree Grabow, Sandra Nolte, Roman Crazzolara, Johann Greil, Martin Ebinger, Michael Abele, Ursula Holzer, Michaela Döring, Johannes H. Schulte, Peter Bader, Paul-Gerhardt Schlegel, Matthias Eyrich, Peter Lang, Thomas Klingebiel, Rupert Handgretinger

**Affiliations:** 1 Department of Haematology and Oncology, University Children’s Hospital, Eberhard Karls University Tuebingen, 72076 Tuebingen, Germany; jonas.rehm97@web.de (J.R.); martin.ebinger@med.uni-tuebingen.de (M.E.); michael.abele@med.uni-tuebingen.de (M.A.); ursula.holzer@med.uni-tuebingen.de (U.H.); michaela.doering@med.uni-tuebingen.de (M.D.); johannes.schulte@med.uni-tuebingen.de (J.H.S.); peter.lang@med.uni-tuebingen.de (P.L.); rupert.handgretinger@med.uni-tuebingen.de (R.H.); 2 Department of Paediatric Oncology and Haematology, University Hospital, University of Cologne, 50937 Cologne, Germany; thorsten.simon@uk-koeln.de (T.S.); barbara.hero@uk-koeln.de (B.H.); 3 Department of Studies and Statistics for Integrated Research and Projects, St Anna Children’s Hospital and Children’s Cancer Research Institute, 1090 Vienna, Austria; ruth.ladenstein@ccri.at; 4 Department of Paediatric Haematology and Oncology, University Medicine Greifswald, 17464 Greifswald, Germany; holger.lode@med.uni-greifswald.de; 5 Division of Childhood Cancer Epidemiology, German Childhood Cancer Registry, Institute of Medical Biostatistics, Epidemiology and Informatics, University Medical Centre of the Johannes Gutenberg University Mainz, 55131 Mainz, Germany; desiree.grabow@uni-mainz.de; 6 Health Economics Unit, Centre for Health Policy, The University of Melbourne, Melbourne, VIC 3010, Australia; sandra.nolte@monash.edu; 7 Eastern Health Clinical School, Faculty of Medicine, Nursing and Health Sciences, Monash University, Melbourne, VIC 3010, Australia; 8 Department of Paediatrics I, Medical University Innsbruck, 6020 Innsbruck, Austria; roman.crazzolara@tirol-kliniken.at; 9 Department of Paediatric Oncology, Haematology and Immunology, University Hospital Heidelberg, 69120 Heidelberg, Germany; johann.greil@med.uni-heidelberg.de; 10 Division for Stem Cell Transplantation and Immunology, Department of Paediatrics, University Hospital, Goethe University, 60590 Frankfurt, Germany; peter.bader@unimedizin-ffm.de (P.B.); thomas.klingebiel@kinderkrebs-frankfurt.de (T.K.); 11 Department of Paediatric Haematology and Oncology, University Children’s Hospital, KIONET Center, 97080 Wuerzburg, Germany; schlegel_p@ukw.de (P.-G.S.); eyrich_m@ukw.de (M.E.)

**Keywords:** neuroblastoma, immunotherapy, pediatric oncology, long-term follow-up

## Abstract

Background: The incorporation of anti-GD2 antibodies such as ch14.18/SP2/0 into the multimodal treatment of high-risk neuroblastoma (HR-NB) patients has improved their outcomes. As studies assessing the long-term outcomes, long-term sequelae, and health-related quality of life (HRQoL) of this treatment are limited, this retrospective analysis aimed to explore these. Patients and Methods: Between 1991 and 2002, 65 children received a multimodal treatment, including ch14.18/SP2/0, for primary HR-NB. All received chemotherapy according to the NB90/NB97 trial, 51 received high-dose chemotherapy, and all received ch14.18/SP2/0 treatment. We analyzed the long-term sequelae and HRQoL (EORTC QLQ-C30), and evaluated overall and event-free survival (OS/EFS). Results: Twenty-five survivors were evaluated for HRQoL and long-term effects. All reported long-term sequelae, including ototoxicity in 16/25 (64%), cardiac toxicity in 6/25 (24%), and endocrine toxicity in 19/25 (76%) patients. Chronic diarrhea was reported in 20% of female patients. Seven patients developed autoimmune diseases. HRQoL scores were better across multiple scales than those of the matched German general population. Twenty-five-year OS and EFS were 50.8% (95% confidence interval: 31–55) and 43% (30.1–55.3), with 33 (50.8%) long-term survivors. Thirty-two patients died: 28 (43.1%) because of progression/relapse and 4 (6.2%) because of secondary neoplasms. Conclusions: Multimodal treatment, including ch14.18/SP2/0, can achieve long-term survival in HR-NB patients, with a substantial proportion of survivors reporting better HRQoL compared to the general population. All patients reported long-term side effects mostly attributable to chemotherapy and radiotherapy. The relatively high prevalence of autoimmune diseases and persistent diarrhea warrants additional longitudinal research on individuals treated with anti-GD2 antibodies.

## Simple Summary

High-risk neuroblastoma is a childhood cancer with poor survival rates despite intensive treatment. Adding anti-GD2 immunotherapy to standard therapies has improved survival, but little is known about the long-term effects (of the anti-GD2-therapy) on and quality of life of survivors. This study followed patients treated with a multimodal treatment including anti-GD2 therapy for over 20 years to assess their outcomes, side effects, and quality of life. While many survivors experienced long-term health issues, such as hearing loss, endocrine problems, and autoimmune diseases, their overall quality of life was often better than that of the general population. This research provides valuable insights into the long-term impact of anti-GD2 therapy, helping to guide future treatments and improve outcomes for children with high-risk neuroblastoma. In addition, the findings highlight the need for lifelong medical care to manage late effects and improve treatment strategies.

## 1. Introduction

Neuroblastoma is the most common pediatric extracranial cancer, and accounts for 15% of childhood cancer-related deaths [1]. About half of the patients show a high-risk phenotype (HR-NB), characterized by metastatic disease in patients > 18 months of age or the amplification of MYCN (MYCNA) [1,2]. The 5-year survival rate of patients with HR-NB is approximately 50%, and immunotherapeutic approaches have been implemented to improve outcomes [1,3]. Targeting the disialoganglioside 2 (GD2) with the human–mouse chimeric antibody ch14.18/SP/2/0 (dinutuximab) improves event-free survival (EFS) and overall survival (OS) as post-consolidation therapy for primary HR-NB patients [4]; however, with a longer follow-up, the improvement in EFS and OS associated with immunotherapy decreased due to late relapses [5]. The acute side effects of immunotherapy have been well described; however, there are no reports of the specific late side effects and health-related quality of life (HRQoL) after this therapy [6,7]. In a single-center study, the number of late effects in patients receiving immunotherapy did not differ from that in other neuroblastoma survivors; however, the side effects were not studied in detail, and the median follow-up time of 5.4 years was relatively short [8].

Here, we report a long-term clinical study of 65 HR-NB patients treated according to the German national trials NB90/NB97 between January 1989 and March 2002 with post-consolidation treatment with ch14.18/SP2/0. Long-term survivors participated in a questionnaire-based survey to determine the proportion of patients with severe toxicity and its impact on HRQoL.

## 2. Materials and Methods

Patients received treatment between November 1991 and July 2002 at Tuebingen University Children’s Hospital, Germany. The eligibility criteria were as follows: (1) age at diagnosis of ≥ 1 year, (2) treatment between 1989 and 2002, (3) stage-4 disease or stage-3 neuroblastoma with MYCNA according to the International Neuroblastoma Staging System (INSS) criteria, and (4) treatment with ch14.18/SP2/0 in post-consolidation [9].

**Figure 1:**
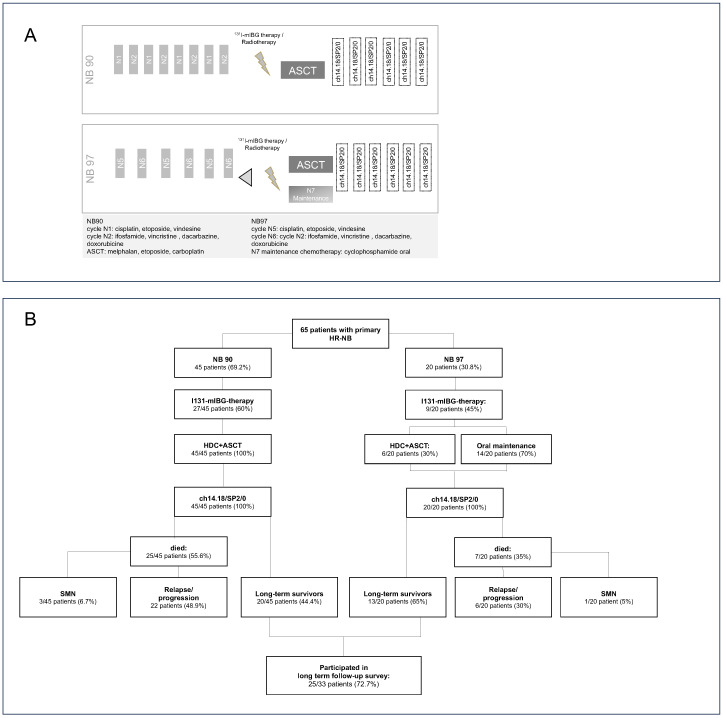
(**A**) Overview of the therapy: In the NB90 trial, high-dose chemotherapy (HDC) with autologous stem cell transplantation was optional; however, all patients in the present cohort received HDC. The NB97 trial was a prospective randomized trial comparing HDC with oral maintenance therapy consisting of four cycles of oral cyclophosphamide for days 1–8 every 28 days. ASCT: autologous stem cell transplantation. (**B**) Details of the study population: 25/33 long-term survivors participated in the long-term survey; the remaining 8 patients did not participate for personal reasons. Nevertheless, the follow-up was updated for these patients. HR-NB: high-risk neuroblastoma. NB90: induction therapy according to German NB90 trial. NB97: induction therapy according to the German NB97 trial. mIBG: iodine meta-iodobenzylguanidine. HDC: high-dose chemotherapy. ASCT: autologous stem cell transplantation. ch14.18/SP2/0: treatment with the chimeric anti-GD2 antibody ch.14.18/SP2/0. SMN: secondary malignancy.

All patients received induction chemotherapy according to the NB90 and NB97 trials [10]. NB90/NB97 consist of alternate chemotherapy cycles including cisplatin, etoposide, vindesine, vincristine, dacarbazine, ifosfamide, and doxorubicin. In NB97, the etoposide dose was reduced by 20%, the doxorubicin infusion time was modified from 48 h to 4 h on two days, and the total number of chemotherapy cycles was reduced from 8 to 6 (Figure 1A).

In NB90, high-dose chemotherapy (HDC) with autologous stem cell transplantation (ASCT) was optional; however, as the Tuebingen Children’s Hospital was a referral center for ASCT, all patients received HDC. The NB97 trial was a randomized trial comparing HDC with four cycles of oral cyclophosphamide for days 1–8 every 28 days.

Autologous peripheral blood stem cells (PBSCs) were collected as previously described [11]. As a purging procedure, CD34+-positive selection was performed [12]. The HDC consisted of melphalan, etoposide, and carboplatin. Furthermore, 131-I-mlBG therapy was recommended for patients with residual meta-iodobenzylguanidine (mIBG)-positive disease at the end of induction and given prior to high-dose chemotherapy [13]. Radiotherapy (36–40 Gray) of residual primary tumors was administered before immunotherapy.

Before immunotherapy, patients had to achieve at least partial remission without a history of progressive disease. The time from stem cell infusion to enrolment was <100 days. Immunotherapy consisted of 5 cycles of ch14.18/SP2/0, 20 mg/m^2^/day, for 5 days, given every four weeks. ch14.18/SP2/0 (Repligen Inc., Needham, MA, USA; BioInvent International, Lund, Sweden) was infused over eight hours each day. For patients who showed tumor response but still had measurable disease, the number of antibody cycles was increased according to the physicians’ discretion. No additional cytokines were given. In patients who saw relapse or progression during the antibody treatment or severe hypersensitivity reactions, antibody therapy was discontinued.

All patients were serially evaluated for tumor response by using bone marrow aspirates/biopsies, mIBG scintigraphy, and CT/MRI scans at increasing time intervals for ten years after therapy.

All long-term survivors were initially contacted by phone and letter for long-term follow-up, and were asked to participate in a questionnaire-based assessment of the long-term side effects and HRQoL. Personal contact data were obtained from the German Childhood Cancer Registry [14].

The questionnaire consisted of two parts. The first part collected information on the participants’ sociodemographic characteristics, such as marital status, educational attainment, professional activity, and closed as well as open questions regarding long-term side effects, secondary diseases, and malignancies. The first part of the questionnaire was used as a screening tool to enable the dedicated evaluation of the long-term side effects in a subsequent step. Patient responses were validated and specified by using medical reports and assessments if necessary.

Late effects were defined as unresolved chronic diseases, symptoms, and toxicities, with relevant medical histories, such as second malignancy and major surgery, recorded. The second part of the questionnaire included the German version of the European Organization for Research and Treatment of Cancer (EORTC) Quality of Life Questionnaire Core 30 (EORTC QLQ-C30), version 3 [15,16]. This encompassed five functional items (physical, emotional, social, role, and cognitive), nine symptom items (fatigue, nausea/vomiting, pain, dyspnea, insomnia, appetite, constipation, diarrhea, and financial difficulties), and one item assessing global health status/overall quality of life (QoL). The EORTC QLQ-C30 used a 4-point scale from “Not at all” to “Very much”, except for global health status/overall QoL, which was rated on a 7-point scale from “Very poor” to “Excellent”. The recall period is “During the past week”, except for the first five physical function items, which specify no recall period. Patients with higher therapeutic exposure due to relapse or disease progression were excluded from the HRQoL and long-term late effect survey. The study obtained local ethics approval (Nr.: 921/2020BO2) and was approved by national regulatory authorities and review boards. Informed consent was obtained from legal guardians and/or patients.

### Statistical Analysis

EFS and OS were estimated by using the Kaplan–Meier method from the date of first diagnosis. EFS was defined as the duration from diagnosis to the first occurrence of relapse, progression, secondary cancer, death, or last contact. OS was measured from diagnosis to death or last contact. For the HRQoL assessment, normative data from the German population were used to interpret patients’ EORTC QLQ-C30 scores [17,18]. The statistical significance of score differences between neuroblastoma survivors and the German population was not assessed due to the small sample sizes; however, differences of >10 points were considered clinically relevant [19]. SPSS^®^ v27 and GraphPad^®^ Prism9 were used for statistical analysis.

## 3. Results

### 3.1. Patient Characteristics and Treatment Regimes

Sixty-five patients with HR-NB diagnosed between January 1989 and September 2002 were analyzed: 39 (60%) were male, and 26 (40%) were female. The median age at diagnosis was 3.2 years (range of 0.3–17.7 years). All but one patient had INSS stage-4 disease, with one patient having MYCNA stage-3 neuroblastoma [9] (Table 1).

Fifty-one of the sixty-five patients (78.5%) received consolidating HDC and ASCT. In the NB97 trial, 14/20 patients (70%) received four cycles of maintenance therapy instead of HDC. Thirty-six patients (55.4%) received 131-I-mlBG therapy with a median activity of 0.55 GBq/kg (Figure 1B).

Post-consolidation immunotherapy with ch14.18/SP2/0 was given to all patients, starting at a mean of 6.5 weeks (range of 6–8 weeks) after HDC or maintenance chemotherapy. The median number of treatment cycles with ch14.18/SP2/0 was five (range of one–nine). Acute toxicities included pain, fever, and fluid retention, as previously described [19].

### 3.2. Survival

The median follow-up from diagnosis was 7.9 years (range of 1–32.2 years), and in long-term survivors it was 24.6 years.

**Figure 2:**
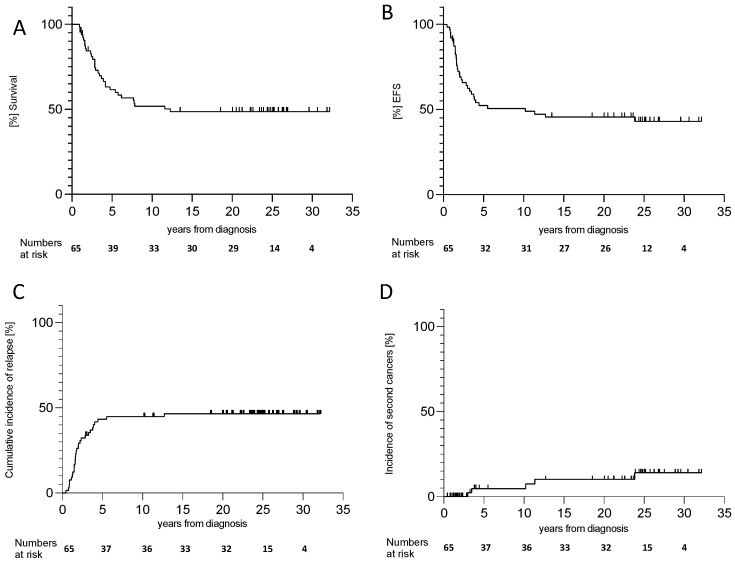
Overall survival (**A**), event-free survival (**B**), cumulative incidence of relapse (**C**), and incidence of secondary cancers (**D**) of the whole cohort.

The median survival was 12.3 years; 10-, 20-, and 30-year EFS was 50.5% (95% confidence interval [CI]: 37.3–62.1), 45.5% (95%CI: 32.7–57.4), and 43% (95%CI: 30.1–55.3), respectively; and 10-, 20-, and 30-year OS was 51.9% (95%CI: 38.9–63.5), 50.8% (95%CI: 35.7–60.3), and 50.8% (95%CI: 35.7–60.3), respectively (Figure 2A,B). Thirty-one patients relapsed or progressed, of whom twenty-eight (90.3%) died (Figure 1B).

Relapse or progression occurred within a median of nine months after the start of the antibody treatment; relapses > 3 years after the end of therapy occurred in four patients. The cumulative incidence of relapse was 43.3% (95%CI: 32.3–56.2) at five, 44.9% (95%CI: 33.7–57.8) at ten, and 46.5% (95%CI: 35.2–59.3) at twenty years (Figure 2C). No therapy-related mortality occurred during the antibody treatment.

### 3.3. Secondary Malignancy (SMN)

Four patients developed fatal secondary malignant neoplasms (SMNs): two developed myelodysplastic syndromes 2 and 10 years post-neuroblastoma diagnosis, one developed neuroendocrine signet cell carcinoma 11.3 years later, and one developed glioblastoma 3.5 years post-therapy. Three patients survived: one was diagnosed with a malignant peripheral nerve sheath tumor (MPNST) in the chest, 24 years after the NB diagnosis; another long-term survivor developed colon carcinoma; and one patient developed a basalioma in the radiotherapy field. No tumor predisposition was found in patients with MPNST or colon carcinoma. The cumulative incidence of SMN was 4.7% (95%CI: 1.2–17.5) at 5 years, 4.7% (95%CI: 1.2–17.5) at 10 years, and 14.0% (95%CI: 5.9–31.1) at 25 years (Figure 2D). Four of the six patients with SMNs received 131-I-mIBG therapy, and three developed SMNs in the radiation area. Benign tumors are listed in Table 2.

### 3.4. Treatment of Neuroblastoma Relapse or SMN in Long-Term Survivors

One patient with NB relapse underwent reinduction chemotherapy within the RIST trial, consolidation with haploidentical stem cell transplantation, and ch14.18/CHO as part of a clinical trial [20,21]. Another patient with local relapse received radiotherapy and additional ch14.18/SP2.0 treatment, while a third patient with progressive disease underwent surgery, radiotherapy, and further antibody treatment. These patients were excluded from the HRQoL and late effect analysis.

Survivors with SMN underwent resection of their tumors without radiotherapy or systemic therapy.

### 3.5. Late Effects

In all evaluated long-term survivors, one or more long-term side effects occurred, with a median number of five per patient (range: 1–18).

The most common long-term effects were ototoxicity (*n* = 16), hypothyroidism (*n* = 8), multiple melanocytic nevi (*n* = 12), and dental abnormalities (*n* = 9). Neurological and psychiatric problems were reported in 12 patients (48%). Height/weight z-scores were less than −1.7 in three patients (12%). Seven patients developed autoimmune diseases: Hashimoto thyroiditis (*n* = 2; two and three years after therapy), diabetes mellitus (*n* = 1; 4.5 years after therapy), inflammatory bowel disease (*n* = 3; 1.5, 3, and 6 years after therapy), and chronic thrombocytopenia (*n* = 1; three months after therapy, meanwhile resolved). Notably, no long-term neuroimmunological complications have been reported to date (Table 2).

### 3.6. Sociodemographic Results and HRQoL

Among the 33 long-term survivors, 18 were male (54.5%) and 15 were female (45.5%), with a median age of 27 years (range of 20–44 years). Updated sociodemographic and HRQoL data were provided by 25 survivors. All 25 had at least a secondary education; 10 were enrolled in or had graduated from a university. Three patients (two female, one male) experienced uncomplicated childbirths (Table 1). The HRQoL assessment results are detailed in Table 3. Male neuroblastoma survivors exhibited clinically significant differences compared to the German general population in 10 of the 15 EORTC QLQ-C30 items. They had higher scores in all five functioning items, global health status/QoL, and lower (better) symptom scores for fatigue, pain, dyspnea, and insomnia. Female survivors showed significant differences in seven items, with higher scores in physical and role functioning as well as global health status/QoL, and lower symptom scores for fatigue, pain, and insomnia; however, female survivors had higher (worse) scores for diarrhea compared to the general population.

## 4. Discussion

The disialoganglioside GD2 is highly expressed on neuroblastoma cells, while its presence on normal tissues is minimal [23]. Anti-GD2 monoclonal antibodies exert their effects through mechanisms such as antibody-dependent cell-mediated cytotoxicity (ADCC) and complement-dependent cytotoxicity (CDC) [24]. Several anti-GD2 antibodies have been tested in clinical trials, including ch14.18. This antibody is a chimeric human/mouse construct, combining variable regions from the murine anti-GD2 antibody 14G2a with constant regions from a human IgG1 molecule. [4,19] It has been shown that immunotherapy with ch14.18 significantly improves outcomes when added to post-consolidation therapy and has become the standard treatment for HR-NB patients [4]; however, knowledge regarding the long-term side effects of anti-GD2 therapy is limited.

Here, we report a long-term update of a cohort of 65 high-risk neuroblastoma patients treated between 01/1989 and 03/2004. With a median follow-up of 24.6 years for survivors, this is the longest follow-up of high-risk neuroblastoma patients treated with a multimodal treatment including ch14.18/SP2/0 or any anti-GD2-directed immunotherapy. In our cohort, the 25-year OS and EFS rates were 50.8% and 43%, respectively. These outcomes are comparable to those reported in the follow-up of COG ANBL0032, with a 5-year EFS of 56.6% and 15-year EFS of approximately 50%. [5]

Our results highlight the importance of lifelong medical care for intensively treated patients to detect and treat late effects. Several analyses have assessed the long-term toxicities in HR-NB survivors treated without immunotherapy, with reports indicating a high prevalence of late effects related to the endocrine system, ototoxicity, and subsequent malignancy [25]. In our cohort, late effects occurred at a comparable frequency. Although anti-GD2 antibodies can cause central nervous system (CNS) toxicity and cross-react with nerve fibers, we observed no long-term neuroimmunological complications in this cohort; however, seven patients developed autoimmune diseases, including inflammatory bowel disease in three and Hashimoto thyroiditis in two patients, which could be attributed to antibody treatment. The induction of autoimmune disease has been reported for several other monoclonal antibodies, such as autoimmune colitis with CTLA4-specific antibodies or autoimmune thyroid disease in patients treated with alemtuzumab [26,27]. A pathogenic relationship between autoimmune effects and anti-GD2 treatment has not been established yet, and these side effects need to be explored further. Interestingly, male neuroblastoma survivors did not report constipation, in contrast to both female patients and the general population.

HR-NB survivors are at an increased risk of psychological problems and impaired QoL, with an increased prevalence of anxiety/depression, attention deficits, peer conflict/social withdrawal, and antisocial behavior, comparable to other solid-tumor survivors [28,29]. In contrast, we found that the general HRQoL in our cohort of neuroblastoma survivors was close to the average, with several scales showing better HRQoL than the normative data obtained from the German general population. This observation was especially true for male survivors, who showed superior HRQoL in 10 of the 15 items, but also for female survivors, who showed some clinically relevant differences compared to the general population. Given that we only had a relatively small sample size, we cannot overinterpret these data; however, the trend of showing better HRQoL compared with the norm data was striking. One reason for the observed differences could be phenomena such as the disability paradox or response shift, a known phenomenon in patients who have experienced severe illnesses and/or other significant life events [30,31]. The only observation in which our cohort showed clinically relevant differences in favor of the general population was diarrhea in females. Chronic diarrhea is a well-known issue in patients with NB, and is often associated with extensive abdominal surgery [32]. Anti-GD2 antibody therapy can acutely worsen this symptom, perhaps due to antibody binding to the autonomic nerve fibers [32]. The persistence of this symptom, even decades after therapy cessation, as reported here, highlights the need for further investigation of its underlying mechanisms.

The incidence of SMN in our cohort was higher than that in previously published cohorts of neuroblastoma survivors, with a reported 15-year cumulative incidence among HR-NB survivors of 5.8% for malignant neoplasms [25]. We assume that this could be related to intense NB90 induction chemotherapy and the large number of patients exposed to I131-mIBG therapy.

The therapeutic exposures in this cohort were relatively similar to those of therapy, which is currently recommended for patients with HR-NB in Europe, and the cohort was homogeneous in terms of risk factors and therapeutic exposure [33]. Nevertheless, the retrospective non-randomized design and the fact that patients were treated over a relatively long time period in a single center are limitations of this study. In addition, we cannot rule out the fact that our cohort is biased, as only 13.8% of patients had MYCNA, and a large proportion of patients received I131-mIBG therapy, which could have positively influenced outcomes.

## 5. Conclusions

In summary, this study provides insights into the long-term effects of a multimodal treatment including anti-GD2 therapy in patients with HR-NB. In the present cohort of patients, a noticeable rate of autoimmune disease and chronic diarrhea was reported, which could be attributed to antibody treatment. Despite several long-term side effects, a substantial proportion of survivors reported even better HRQoL than the general population norm data. With improved survival rates and the inclusion of anti-GD2 treatment in various treatment compounds, the continued monitoring of long-term survivors is necessary, and future studies should focus on the impact of anti-GD2 therapy on potential long-term side effects. Future therapy regimens should also aim to reduce long-term side effects and secondary malignancy.

## Figures and Tables

**Table 1:** Patient characteristics, at diagnosis and at the latest follow-up.

Variable	N (%)/Range
Patient Characteristics at Diagnosis	
Sex	
Male	39 (60%)
Female	26 (40%) ^1^
Median age at diagnosis (range)	3.2 years (0.3–17.7 years)
INSS Stage at Diagnosis	
3	1 (1.5%) ^2^
4	64 (98.5%)
Primary	
Abdominal	51 (78.5%)
Cervical/thoracic	10 (15.4%)
Unknown	3 (4.6%)
Tumor MYCN Amplification Status	
Amplified	9 (13.8%)
Non-amplified	50 (76.9%)
Unknown	6 (9.2%)
Bone Marrow Involvement	
Yes	48 (73.8%)
No	10 (15.4%)
Unknown	7 (10.8%)
Initial Treatment	
Chemotherapy	65 (100%)
NB90	45 (69.2%)
NB97	20 (30.8%)
Radiotherapy	36 (55.4%)
131-I-mIBG therapy	36 (55.4%)
High-dose chemotherapy with autologous stem cell transplantation	51 (78.5%)
Relapse Treatment (Survivors)	
Chemotherapy	1/3 (33%)
Surgery	1/3 (33%)
Radiotherapy	2/3 (66%)
Anti-GD2 antibody treatment	3/3 (100%)
Haploidentical stem cell transplantation + ch14.18/CHO + IL2	1/3 (33%)
Patient Characteristics at Follow-Up	
Age at latest follow-up, median (range)	27 years (20–44 years)
Follow-up of all patients, median (range)	7.9 years (1–32.2 years)
Follow-up of long-term survivors, median (range)	24.6 years (13.5–32.2 years)
Highest Level of Education at the Time of the Latest Follow-Up	
Secondary education	4 (16%)
High school degree, including enrollment at a university	16 (64%)
University degree	5 (20%)
Childbirth	3 (4.6%)

INSS: international neuroblastoma staging system. ^1^ One patient listed as female identifies as male and underwent sex-affirming surgery. Sex is listed according to sex assigned at birth and diagnosis (female). ^2^ INSS stage 3 with MYCN amplification.

**Table 2:** Late effects in neuroblastoma survivors.

Late Effects	*n* (%)
Any	25 (100%)
Two or more	17 (68%)
Height/weight score < −1.7	3 (12%)
Endocrine	24 (96%)
Hypothyroidism	8 (32%)
Hashimoto thyroiditis	2 (8%)
Growth hormone deficiency (treated)	3 (12%)
Gonadal failure	6 (24%)
Infertility	4 (16%)
Diabetes mellitus	1 (4%)
Pancreatic insufficiency	1 (4%)
Gastrointestinal	3 (12%)
Inflammatory bowel disease	3 (12%)
Chronic gastritis/duodenitis	4 (16%)
Ear	16 (64%)
Hearing loss (any)	16 (64%)
Eye	7 (28%)
Myopia	4 (16%)
Horner’s syndrome	3 (12%)
Neurologic/psychiatric	12 (48%)
Migraine	3 (12%)
Speech development disorder	3 (12%)
Reading disorder/spelling disorder	3 (12%)
Attention deficit disorder	1 (4%)
Depression	1 (4%)
Borderline personality disorder	1 (4%)
Anxiety	1 (4%)
Neuro-immunological	0
Cardiac	6 (24%)
Left ventricular systolic dysfunction	2 (8%)
Aortic valve insufficiency	1 (4%)
Arterial hypertension	3 (12%)
Blood	3 (12%)
Chronic anemia	1 (4%)
Chronic thrombocytopenia ^1^	1 (4%)
Pulmonary	4 (16%)
Asthma	1 (4%)
Shortness of breath	3 (12%)
Skin	12 (48%)
Melanocytic naevi	12 (48%)
Orthopedic	2 (8%)
Osteoporosis	1 (4%)
Scoliosis	1 (4%)
Renal	3 (12%)
Chronic renal insufficiency	2 (8%)
Polycystic kidney disease	1 (4%)
Teeth	9 (36%)
Dental developmental anomalies	9 (36%)
Benign tumors	10 (40%)
Benign thyroid nodules/adenomas	3 (12%)
Multiple hemangiomas	1 (4%)
Oncocytoma of the kidney	1 (4%)
Multiple angiomyolipomas of the kidney	1 (4%)
Focal nodular hyperplasia (FNH)	1 (4%)
Ovarian fibroma	1 (4%)
Schwannoma lung	1 (4%)
Neurofibroma	1 (4%)
Malignancies	3 (12%)
Basalioma	1 (4%)
MPNST	1 (4%)
Colon carcinoma	1 (4%)

MPNST: malignant peripheral nerve sheath tumor; ^1^ chronic thrombocytopenia resolved approximately four years after the end of antibody treatment and was diagnosed as immune thrombocytopenia.

**Table 3:** EORTC QLQ-C30 scores of male (*n* = 10) and female (*n* = 15) neuroblastoma patients compared with normative data of the German general population.

	Males			Females ^1^		
EORTC QLQ-C30 Questionnaire	NB Patients (*n* = 10)	General Population Norm Data	Mean Difference NB Patients Versus Norm Data	NB Patients (*n* = 15)	General Population Norm Data	Mean Difference NB Patients Versus Norm Data
	M ± SD	M	MD	M ± SD	M	MD
Physical functioning	99.3 ± 2.1	84.3	15.0	93.8 ± 6.4	81.4	12.4
Role functioning	100 ± 0	82.0	18.0	90.0 ± 21.6	79.7	10.3
Emotional functioning	85.8 ± 15.2	74.7	11.1	66.7 ± 21.1	73.1	−6.4
Cognitive functioning	93.3 ± 8.6	82.6	10.7	87.8 ± 14.7	85.2	2.6
Social functioning	95.0 ± 11.2	83.9	11.1	84.4 ± 17.2	85.7	−1.3
Global health status/QoL	89.2 ± 13.6	68.2	21.0	76.1 ± 17.2	65.8	10.3
Fatigue	11.1 ± 11.7	29.1	−18.0	23.0 ± 19.4	33.8	−10.8
Nausea/vomiting	0 ± 0	6.6	−6.6	2.2 ± 5.9	5.5	−3.3
Pain	3.3 ± 7.0	24.3	−21.0	20.0 ± 25.3	30.7	−10.7
Dyspnea	3.3 ± 10.5	18.1	−14.8	13.3 ± 27.6	19.3	−6.0
Insomnia	6.7 ± 14.0	25.4	−18.7	13.3 ± 27.6	29.7	−16.4
Appetite loss	3.3 ± 10.5	9.6	−6.3	4.4 ± 11.7	10.7	−6.3
Constipation	0 ± 0	9.2	−9.2	11.1 ± 20.6	9.9	1.2
Diarrhea	6.7 ± 14.0	11.4	−4.7	20.0 ± 32.8	9.5	10.5
Financial difficulties	13.3 ± 23.3	10.9	2.4	11.1 ± 20.6	11.6	−0.5

^1^ One NB patient listed as female identifies as male and underwent sex-affirming surgery. The patient was grouped according to the sex assigned at birth and NB diagnosis (female). NB: neuroblastoma; MD: mean difference; *n*: sample size; M: mean; and SD: standard deviation. Differences of >10 points are considered to be clinically relevant between NB patients and the general population normative data [22].

## Data Availability

The data that support the findings of this study are available on request from the corresponding author. The data are not publicly available due to privacy restrictions.
